# Rapid Recombination by Cadmium Vacancies in CdTe

**DOI:** 10.1021/acsenergylett.1c00380

**Published:** 2021-03-19

**Authors:** Seán R. Kavanagh, Aron Walsh, David O. Scanlon

**Affiliations:** † Thomas Young Centre and Department of Chemistry, University College London, 20 Gordon Street, London WC1H 0AJ, U.K.; ‡ Thomas Young Centre and Department of Materials, Imperial College London, Exhibition Road, London SW7 2AZ, U.K.; ¶ Department of Materials Science and Engineering, Yonsei University, Seoul 03722, Republic of Korea; § Diamond Light Source Ltd., Diamond House, Harwell Science and Innovation Campus, Didcot, Oxfordshire OX11 0DE, U.K.

## Abstract

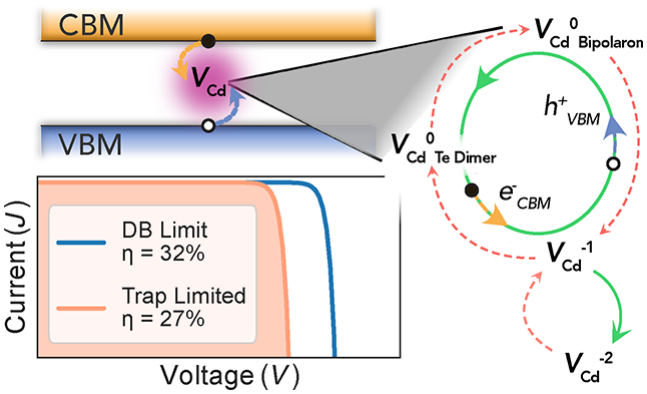

CdTe is currently the largest thin-film photovoltaic technology. Non-radiative electron–hole recombination reduces the solar conversion efficiency from an ideal value of 32% to a current champion performance of 22%. The cadmium vacancy (V_Cd_) is a prominent acceptor species in *p*-type CdTe; however, debate continues regarding its structural and electronic behavior. Using *ab initio* defect techniques, we calculate a negative-U double-acceptor level for V_Cd_, while reproducing the V_Cd_^1–^ hole–polaron, reconciling theoretical predictions with experimental observations. We find the cadmium vacancy facilitates rapid charge-carrier recombination, reducing maximum power-conversion efficiency by over 5% for untreated CdTe—a consequence of tellurium dimerization, metastable structural arrangements, and anharmonic potential energy surfaces for carrier capture.

Cadmium telluride (CdTe) is a well-studied thin-film photovoltaic (PV) absorber, being one of few solar technologies to achieve commercial viability.^1^ Its ideal 1.5 eV electronic band gap and high absorption coefficient have allowed it to reach record light-to-electricity conversion efficiencies of 22.1%.^2−4^ Given that device architectures and large-scale manufacturing procedures have been highly optimized for this technology—a result of several decades of intensive research^2,5^—further reductions in cost will be heavily dependent on improvements in photoconversion efficiency.^1,2,6^ Indeed, under the idealized detailed balance model, CdTe has an upper limit of 32% single-junction PV efficiency (based on its electronic bandgap),^7^ indicating that there is still room for improvement.^6,8−11^

Despite over 70 years of experimental and theoretical research,^2,12−19^ the defect chemistry of CdTe is still not well understood. The unambiguous identification of the atomistic origins of many experimentally observed spectroscopic signatures remains elusive. Only through clear understanding of defect behavior can strategies be devised to avoid and/or mitigate their deleterious effects on device performance.^20−23^

At present, market-leading CdTe solar cells employ a Te-rich *p*-type CdTe absorber layer, favoring the formation of Cd vacancies. Indeed, *undoped* CdTe grown from the melt is typically found to exhibit native *p*-type behavior,^14^ which has often been attributed to the presence of vacancies in the Cd sub-lattice (and/or Te-on-Cd antisites).^18^ However, the exact origin of this low intrinsic *p*-type conductivity is still not well understood, with difficulties in definitive measurements^14−16,24^ and discrepancies between models and observations.^2,25−28^ While there is consensus that the cadmium vacancy (V_Cd_) is an important acceptor species in CdTe, strong debate has endured regarding its structural and electronic behavior.^2,14,18,26−32^

The ability of modern theoretical approaches to accurately describe defect behavior is well established.^20,33,35^ The use of a sufficiently accurate Hamiltonian is essential for reliable predictions. For CdTe, using a screened hybrid Density Functional Theory (DFT) functional with spin–orbit coupling (HSE+SOC), we find that the room-temperature experimental bandgap of 1.5 eV is reproduced at a Hartree–Fock exchange fraction α_exx_ = 34.5%, a value which also reproduces the experimental lattice constant to within 1% (see Supporting Information). For consistency, this model was employed in all structural optimizations and electronic calculations.

## Cadmium Vacancy: Equilibrium Structures

**Figure 1:**
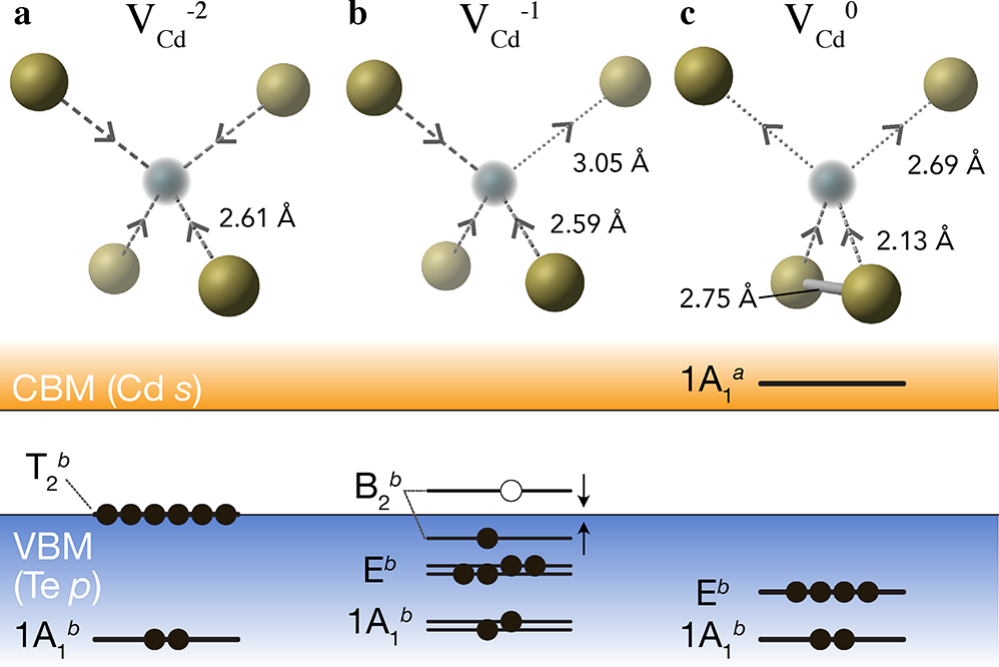
(Top) Ground-state structures of the cadmium vacancy in the double-negative (V_Cd_^2–^, a), single-negative (V_Cd_^1–^, b), and neutral (V_Cd_^0^, c) charge states. Tellurium atoms are shown in gold and cadmium vacancy center-of-mass in ocean blue, with each unique Te–V_Cd_ distance labeled. (Bottom) The corresponding electron energy level diagrams at the Γ point, with character symmetry labels. Superscripts b and a refer to bonding- and antibonding-type interactions, respectively.

The first step in any theoretical investigation of solid-state defects is the determination of their equilibrium structures. CdTe crystallizes in the zinc-blende structure (space group *F*4̅3*m*), thus exhibiting tetrahedral (*T*_*d*_) symmetry at both the Cd and Te sites. The relaxed geometric configurations upon creation of a cadmium vacancy in the neutral (V_Cd_^0^), single-negative (V_Cd_^1–^), and double-negative (V_Cd_^2–^) charge states are shown in Figure 1. Only the double-negative defect retains the original tetrahedral point-group site symmetry, with a contraction of the neighboring Te atoms from the original bond distance of 2.83 Å to 2.61 Å from the vacancy center-of-mass.

The defect site distortions can be rationalized through consideration of the local bonding behavior in a molecular orbital model.^36,37^ Removal of a Cd atom (and its two valence electrons) to create a vacancy results in a fully occupied A_1_ electron level and a two-thirds occupied T_2_ level at the Fermi level, arising from the tetrahedral coordination of Te sp^3^-hybrid orbitals. In the double-negative case (V_Cd_^2–^), the T_2_ level becomes fully occupied, and thus tetrahedral point symmetry is maintained (Figure 1a), with the Te atoms moving closer to the vacancy site to allow for greater hybridization between dangling bonds.

**Figure 2:**
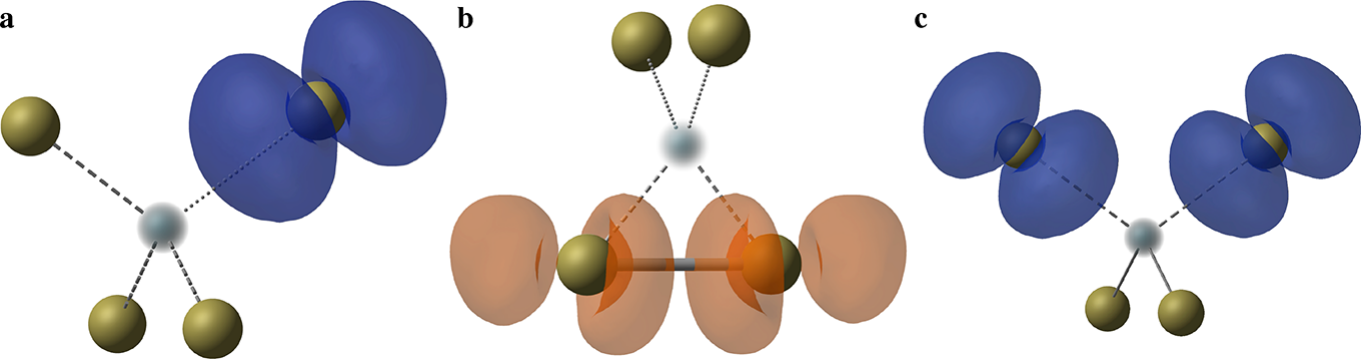
Spin-polarized charge-density isosurfaces of the localized hole polaron for the singly charged defect (V_Cd_^1–^, a), the unoccupied antibonding Te dimer state in the neutral vacancy (V_Cd_^0^, b), and the metastable high-spin bipolaron state for the neutral vacancy (V_Cd,Bipolaron_^0^, c). Tellurium atoms are shown in gold and cadmium vacancy center-of-mass in ocean blue. Isovalues are set to 0.006 e/Å^3^ for the polarons (a, c) and 0.002 e/Å^3^ for the dimer state (b).

For the singly charged vacancy, the 5/6 partial occupancy of the T_2_ level is unstable, undergoing a trigonal Jahn–Teller distortion that substantially elongates one of the Te neighbor distances (Figure 1b). In this *C*_3*v*_-symmetry vacancy coordination, a positive hole is strongly localized on the Te atom furthest from the vacancy site, as depicted in Figure 2a, resulting in a paramagnetic defect species. This *C*_3*v*_ polaronic structure of V_Cd_^1–^ was experimentally identified in the 1990s, using electron paramagnetic resonance (EPR),^14,16^ but was only reproduced for the first time in a 2015 theoretical study by Shepidchenko et al.,^38^ using the HSE06 functional. The primary reason why previous *ab initio* works^2,25,28,39−42^ have failed to identify this polaronic ground-state structure for V_Cd_^1–^ is the spurious electron self-interaction and consequent over-delocalization inherent in standard (semi)local DFT functionals.^20,43−45^

In the neutral case, we find that the Cd vacancy undergoes strong local relaxation to a *C*_2*v*_ structural motif, whereby two Te atoms move significantly closer both to the vacancy site and to each other (2.75 Å separation from an initial 4.63 Å) (Figure 1c). This yields a Te dimer arrangement with occupied sp^3^ σ-bonding electronic levels deep in the valence band and unoccupied antibonding states in the conduction band (Figure 2b). Notably, this Te dimerization resembles that observed at low-energy surfaces and grain boundaries in CdTe and has been suggested as a source of fast recombination at these locations.^10,46,47^ Similar metal–metal dimer reconstructions have been noted for neutral *anion* vacancies in the II–VI semiconductors ZnSe and ZnS,^48^ occurring here for the *cation* vacancy in CdTe due to the metalloid character of the Te anion.

**Figure 3:**
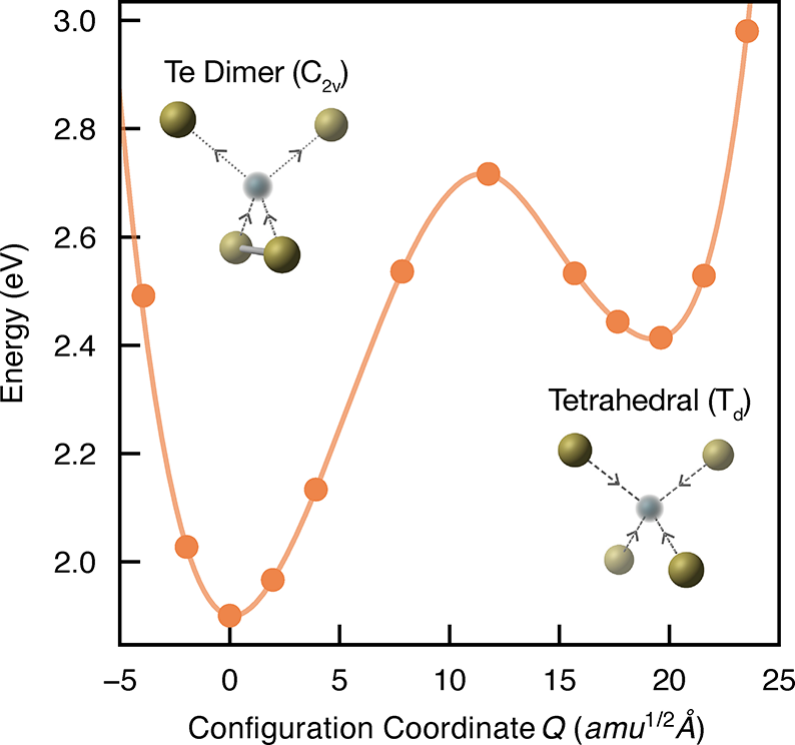
Potential energy surface for V_Cd_^0^ along the configurational path from the “Te dimer” (*Q* = 0 amu^1/2^ Å) to tetrahedral (*Q* ≃ 20 amu^1/2^ Å) arrangement. Filled circles represent the calculated formation energies at a given configuration coordinate, and the solid line is a spline fit. *Q* is given in terms of mass-weighted displacement, and Te-rich conditions (μ_Te_ = 0) are assumed.

**Figure 4:**
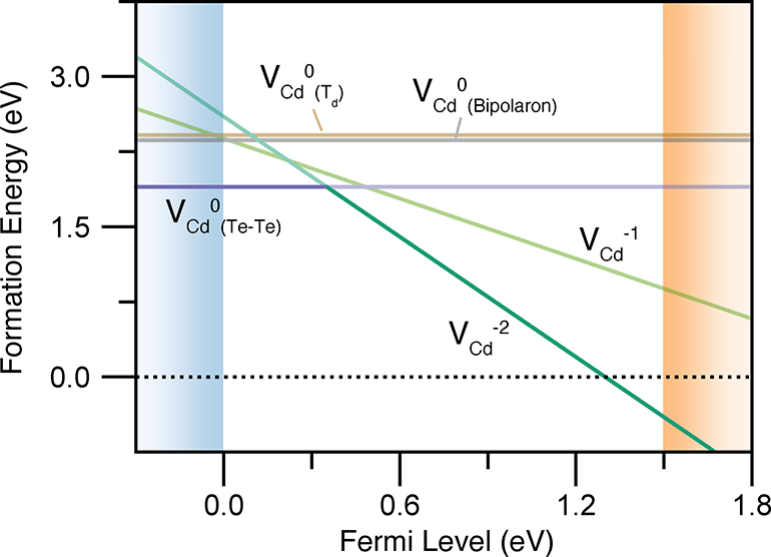
Defect formation energy diagram for the cadmium vacancy in CdTe, under Te-rich conditions (μ_Te_ = 0), with the thermodynamically favored state for a given Fermi level (*E*_F_) shown in saturated color. All locally stable configurations for the neutral vacancy are included.

This atomic reconstruction reduces the vacancy formation energy by 0.52 eV, relative to the tetrahedral solution that has been widely reported^28,39−42,49−51^ (Figures 3 and 4). As with the *C*_3*v*_ Jahn–Teller distortion for V_Cd_^1–^, this Te dimer equilibrium structure of the neutral vacancy has only recently been identified.^18^ The tetrahedral and bipolaron (Figure 2c) configurations are in fact local minima on the defect potential energy surface (PES), as shown in Figures 3, 4, and S7.

The electronic behavior of the Cd vacancy is dramatically affected by Te dimerization, as the singly charged state V_Cd_^1–^ is consequently predicted to be thermodynamically unfavorable across all Fermi energies (Figure 4). Accordingly, the vacancy is predicted to act as a so-called negative-U center,^52,53^ with a single double-acceptor level at 0.35 eV above the valence band maximum (VBM). This is in excellent agreement with experimental reports of a single *thermal* ionization level in the bandgap at 0.3−0.4 eV above the VBM (Table S1).^29,31,32,54−58^ Moreover, negative-U behavior helps to explain apparent discrepancies between experimental reports of Cd vacancy trap levels, as different techniques can measure either the single-charge (2– → 1– and 1– → 0) or double-charge transitions (2– → 0).^59^ The reasons previous theoretical works have not identified this behavior are two-fold: namely, incomplete mapping of the defect potential energy surface (overlooking Te–Te dimerization in V_Cd_^0^) and qualitative errors in lower levels of electronic structure theory (destabilizing localized solutions; viz. the V_Cd_^1–^ small-polaron); see Supporting Information, Section S6, for further discussion.

## Optical Response

The paramagnetic nature of the single negative charge vacancy V_Cd_^1–^ (due to the presence of an odd number of electrons) lends itself to experimental identification through electron spin resonance (ESR/EPR) spectroscopy. In 1993, Emanuelsson et al.^14^ used photo-ESR to identify the *C*_3*v*_ coordination of V_Cd_^1–^, with a localized hole on a Te neighbor as predicted here (Figure 2a). After thermal annealing at 750 °C, they obtained a *p*-type CdTe film with a carrier concentration *p* = 1.2 × 10^17^ cm^–3^, in excellent agreement with our predicted maximum hole concentration of *p* = 4.2 × 10^17^ cm^–3^ at this temperature (based on calculated intrinsic defect formation energies). While V_Cd_^1–^ is never the lowest energy configuration at equilibrium, we find that Cd vacancies do in fact adopt this charge state under high-temperature *p*-type growth conditions, as a consequence of energy minimization within the constraint of charge neutrality (to counteract the large hole concentration).

**Figure 5:**
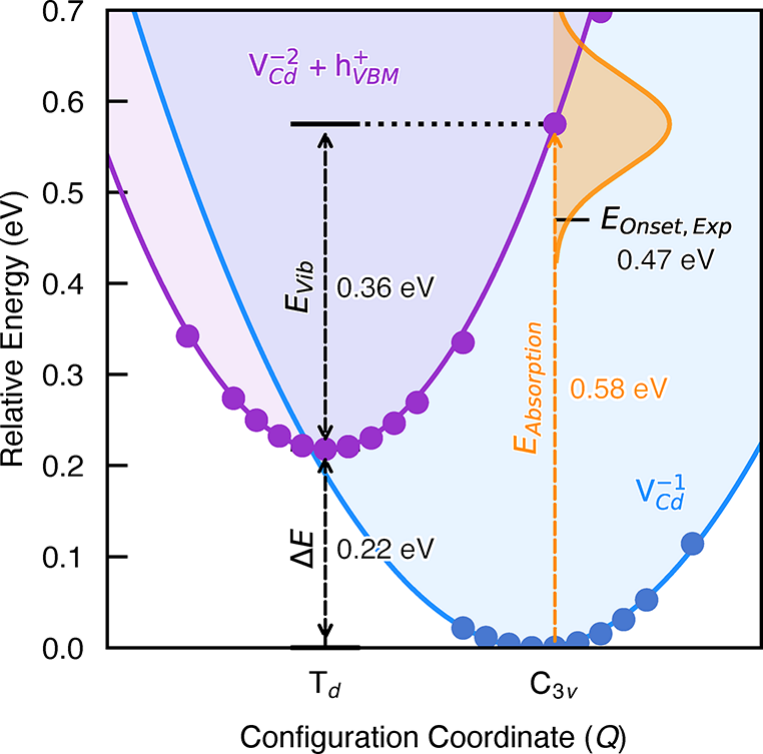
Configuration coordinate diagram for the V_Cd_^1–^ → V_Cd_^2–^ transition, showing the calculated optical excitation (*E*_Absorption_) with vibrational broadening (orange curve), vibrational relaxation (*E*_Vib_), thermodynamic transition (Δ*E*), and experimental absorption onset (*E*_Onset,Exp_) energies. The solid lines are harmonic fits to the DFT energies, represented by filled circles. *X*-axis labels correspond to the defect point-group symmetry.

Emanuelsson et al.^14^ interpreted a decrease in the V_Cd_^1–^ ESR intensity upon irradiation with photons of energy *h*ν > 0.47 eV as the optical excitation of an electron from the valence band to the (−/2−) V_Cd_ level, to produce V_Cd_^2–^ + *h*_VBM_^+^. Using the defect structures obtained in our investigations, we calculate the peak energy of this transition as 0.58 eV, with vibronic coupling estimated to give a Gaussian line shape with a fwhm of 0.12 eV, yielding good agreement with experiment (Figure 5).

## Trap-Mediated Recombination

To determine the non-radiative recombination activity, electron and hole capture coefficients were calculated for each charge state of the defect. This approach, building on the developments of Alkauskas et al.,^60^ uses the CarrierCapture.jl package,^61^ and full details of the calculation procedure are provided in the Supporting Information, Section S8. The PES of the defect is mapped along the structural path (configuration coordinate) *Q* between the equilibrium geometries for a given charge transition, from which nuclear wave function overlaps can be determined via the 1D Schrödinger equation.^60,62^ Electron–phonon coupling is then calculated under static coupling perturbation theory which, in combination with phonon overlaps and scaling factors for charge interaction effects, yields the carrier capture coefficients *C*_*p*/*n*_^*q*^.

**Figure 6:**
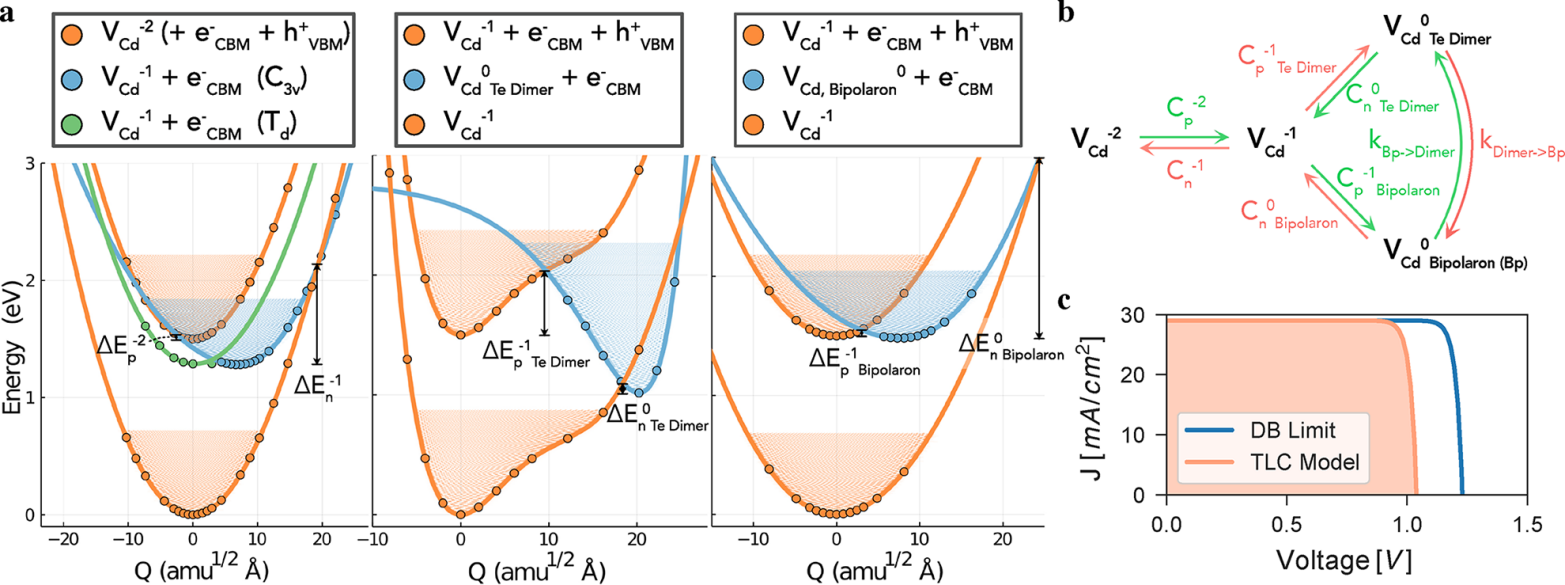
(a) Potential energy surfaces of the (2–/−) (left), (−/0)_Te Dimer_ (center), and (−/0)_Bipolaron_ (right) charge transitions for V_Cd_ in CdTe, with Δ*E*_*p*/*n*_^*q*^ denoting the classical energy barrier to hole/electron capture by a vacancy in charge state *q*. Filled circles represent calculated energies, and the solid lines are best fits to the data. The vibrational wave functions are also shown. *Q* is the configurational coordinate path between equilibrium configurations, given in units of mass-weighted displacement. (b) Schematic of the non-radiative recombination mechanism at the cadmium vacancy, with the dominant (rapid) processes colored green. (c) *J*–*V* curve for an ideal CdTe solar cell, based on the bulk electronic properties and excluding interfacial effects. “TLC” (trap-limited conversion efficiency) refers to a device limited by non-radiative recombination at V_Cd_ (details in text), and “DB” is the detailed balance limit.

The energy surfaces for all in-gap V_Cd_ carrier traps are shown in Figure 6 and the resulting capture coefficients tabulated in the Supporting Information, Section S8. As expected for an acceptor defect with a trap level near the VBM (Figure 4), hole capture is fast while electron capture is slow for the (2–/−) transition, with small and large capture barriers, respectively. For the V_Cd_^1–^ ⇄ V_Cd_^0^ transitions, however, the behavior is drastically different to that predicted by a simple quantum defect model.^63^ First, hole capture is more rapid than expected, due to the ability of V_Cd_^1–^ to transition to the metastable V_Cd,Bipolaron_^0^ configuration, before relaxing to the V_Cd,Te Dimer_^0^ ground state. Second, despite the (−/0)_Te Dimer_ trap level lying over 1 eV below the CBM (Figure 4), typically implying slow electron capture, we in fact find a giant electron capture coefficient. This unusual behavior is a direct result of the anharmonicity of the PESs at this trap center, accompanied by large electron–phonon coupling, through Te dimer formation. These findings provide additional evidence to support Te dimerization at surfaces and grain boundaries in CdTe as a cause of high recombination velocities at these locations.^10,46,47^ Consequently, the (−/0) V_Cd_ charge transition is predicted to facilitate rapid electron–hole recombination, proceeding via the {V_Cd_^1–^ + *e*_CBM_^–^ + *h*_VBM_^+^} → {V_Cd,Bipolaron_^0^ + *e*_CBM_^–^} → {V_Cd,Te Dimer_^0^ + *e*_CBM_^–^} →{V_Cd_^1–^} cycle shown in Figure 6b. Notably, the large capture coefficients for the rapid (green) processes are comparable to the most deleterious extrinsic defects in silicon^64,65^ and the kesterite photovoltaic family.^62,66^ This classifies V_Cd_ as a “killer center” ^67^ and demonstrates the potential impediment of this native defect species to the photovoltaic efficiency of untreated CdTe.

To quantify the effect of this recombination channel on CdTe solar cell performance, we calculate the trap-limited conversion efficiency (TLC),^66^ which incorporates the effects of defect-mediated non-radiative recombination via the Shockley–Read–Hall model.^68^ This allows us to set an upper limit on the achievable photovoltaic efficiency in the presence of defects. As depicted in the current–voltage curve in Figure 6c, we find that cadmium vacancies can significantly reduce the open-circuit voltage (*V*_OC,TLC_ = 1.04 V), minority carrier lifetime (τ_e_ = 29 ns), and thus the maximum achievable photovoltaic efficiency from the ideal 32.1% to 26.7% (for intrinsic *p*-type CdTe processed under typical anneal temperatures of 600 °C in a Te-rich atmosphere, see Supporting Information, Section S8). Due to the large hole concentrations in the *p*-type compound, V_Cd,Te Dimer_^0^ will be the dominant state under steady-state illumination, with electron capture by this defect species representing the rate-limiting step:
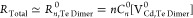
Our prediction is a testament to the importance of Cl treatment, strategic impurity doping, and Cd-rich growth environments in the fabrication of high-efficiency CdTe devices,^9,11,32,34,69−79^ which contribute to the passivation and reduction of cadmium vacancy populations. Notably, the recent achievement of open-circuit voltages surpassing the 1 V threshold for CdTe solar cells by Burst et al.^11^ required a switch to an unorthodox strategy of Cd-rich growth conditions and group V anion doping, reducing the formation of V_Cd_ (and Te_Cd_).

In conclusion, we reconcile several longstanding discrepancies between theoretical predictions and experimental measurements for CdTe, predicting both a single double-acceptor level and the *C*_3*v*_ V_Cd_^1–^ hole–polaron state for the cadmium vacancy in CdTe. An equilibrium population of cadmium vacancies can facilitate rapid recombination of electrons and holes, reducing the maximum achievable power-conversion efficiency under idealized conditions by over 5%, for untreated CdTe. These recombination kinetics primarily arise from both metastable vacancy structures and the Te dimer configuration of V_Cd_^0^ which, in addition to producing negative-U behavior, leads to anharmonic carrier capture PESs. Importantly, these results demonstrate the necessity to include the effects of both metastability and anharmonicity for the accurate calculation of charge-carrier recombination rates in photovoltaic materials.

## Supporting Information Available

Computational methods; supporting notes S1 and S2; Figures S1–S3 and Tables S1–S4, showing bandgap-corrected hybrid DFT functional; bulk electronic structure; vacancy bonding, structural, and electronic analysis, including; discrepancies in theoretical studies; carrier capture model, results, and analysis, experimental identification of tellurium dimerization; defect electronic densities of states; and chemical potentials (PDF)

## Notes

Data produced during this work is freely available at https://zenodo.org/record/4541602.
